# GDE2/GDPD5 in neuroblastoma

**DOI:** 10.18632/oncotarget.14233

**Published:** 2016-12-26

**Authors:** Elisa Matas-Rico, Michiel van Veen, Wouter H. Moolenaar

**Affiliations:** ^1^ Division of Cell Biology, The Netherlands Cancer Institute, Plesmanlaan, Amsterdam, The Netherlands

**Keywords:** differentiation, glycerophosphodiesterase, GPI-anchor, proteoglycan, biomarker, Neuroscience

**Figure 1:**
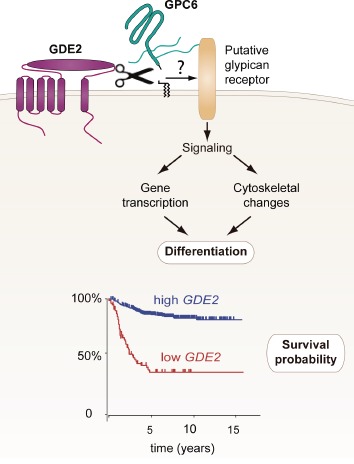
GDE2 cleaves GPI-anchored glypican-6 (GPC6) to signal differentiation of neuroblastoma cells through altered interaction with a putative transmembrane receptor. High GDE2 expression correlates with favorable clinical outcome [4].

Neuroblastoma is an embryonal cancer of early childhood that originates from the developing sympathetic nervous system, usually arising in the adrenal gland. It is the most common malignancy during infancy, accounting for about 10% of childhood cancer mortality, and there is an urgent need for new therapeutic approaches. Neuroblastoma is a highly heterogeneous disease, characterized by relatively few recurrent somatic mutations [1]. In some cases, at very young age, the tumor can undergo spontaneous regression through poorly understood mechanisms [2]. In many cases, however, neuroblastoma progresses into a high-risk metastatic disease. Neuroblastoma originates from the neural crest and is characterized by aberrant growth and impaired neuronal differentiation of immature neuroblasts. In general, patient survival depends on the degree of neuronal differentiation in the primary tumors and is inversely correlated with a motile phenotype. Some high-risk neuroblastomas are associated with mutations in Rho GTPase pathway genes that normally regulate the differentiated phenotype and are implicated in neuritogenesis and cell motility [3]. Unfortunately, treatment options are very limited for high-risk neuroblastoma. To identify new therapeutic targets, it will be essential to gain a better understanding of neuroblastoma differentiation at the molecular level. Indent in a recent study, we have uncovered a previously unknown mechanism of neuroblastoma differentiation with prognostic significance [4]. The new mechanism involves the action of GDE2 (encoded by *GDPD5*), a transmembrane glycosylphosphatidylinositol (GPI)- specific glycerophosphodiesterase and member of a larger GDE family [5]. GDE2 was earlier reported to promote the differentiation of spinal motor neurons by cleaving a GPI-anchored Notch ligand regulator, resulting in down-regulation of Notch signaling in adjacent neural progenitors [6]. In our study, GDE2 induced neuroblastoma cell differentiation in a cell-autonomous manner through cleavage and release of a GPI-anchored heparan sulfate proteoglycan, termed glypican-6 (GPC6). By releasing GPC6, GDE2 was found to initiate a cell-intrinsic differentiation program involving altered Rac/ Rho activity and transcription of multiple differentiation-associated genes. Phenotypically, GDE2-induced GPC6 release led to increased cell-matrix adhesion, cell spreading, neurite outgrowth, blocked neurite retraction, and reduced cell motility [4], as illustrated in Figure 1. Strikingly, high expression of *GDPD5* was strongly associated with favorable clinical outcome, while low *GDPD5* expression correlated with poor outcome in independent patient cohorts [4] (Figure 1). Conversely, high expression of GDE2 substrate GPC6 was associated with poor prognosis.

*GDPD5* maps to chromosome 11q13, a region often showing loss of heterozygosity in high-risk neuroblastoma. Based on these results, *GDPD5* would qualify as a potential tumor suppressor gene. However, no mutations or deletions were detected in the tumor samples analyzed [4]. Furthermore, *GDPD5* expression in patients with an 11q deletion was similar to those with a normal chromosome 11, neither was there an inverse correlation with amplification of the *MYCN* oncogene. While the patient survival data are consistent with the cell-based results – namely that GDE2 suppresses the malignant phenotype through GPC6 release - it remains possible that GDE2 cleaves additional GPI-anchored substrates whose functional loss may contribute to favorable outcome in neuroblastoma. It will therefore be important to establish the substrate selectivity of GDE2 in further detail.

The major outstanding question is how GPI-anchor cleavage of a heparan sulfate proteoglycan, GPC6, triggers multiple intracellular signaling events to alter cellular phenotype. GPI-anchoring of proteins into the outer leaflet of the plasma membrane represents a complex post-translational modification, and its biological function has remained obscure for decades. Being GPI-anchored, glypicans lack intrinsic signaling capability. Instead, they are capable of recruiting and distributing growth factors, dictated by the structure of their glycosaminoglycan chains. GDE2-induced glypican release could thus result in growth factor redistribution with potentially important consequences for receptor activation and downstream signaling pathways. Perhaps more relevant in the present context, cell-surface proteoglycans can also serve as membrane-tethered ligands in their own right by directly interacting with transmembrane receptors, such as type II receptor tyrosine phosphatases (RPTPs), as we discussed [4]. Therefore, it is tempting to speculate that glypican release might alter the activation status of one or more transmembrane RPTPs and trigger phosphotyrosine-based signaling events to impact neuronal differentiation, a scenario that warrants further study. Whatever the nature of the putative glypican-responsive receptor(s), the finding that a cell-intrinsic phosphodiesterase such as GDE2 selectively hydrolyzes GPI anchors to modulate signaling pathways and alter cell behavior sheds new light on the biological function of the once mysterious GPI anchors [4, 6, 7]. It remains to be determined whether GDE2 functions as a GPI-specific phospholipase C or D to cleave its substrates.

From a clinical perspective, the new findings suggest that activation of the GDE2 signaling pathway by pharmacological means might be of benefit to patients having low GDE2 expression levels. As an ecto-phosphodiesterase, GDE2 is in theory a convenient drug target. However, it will be quite challenging to develop drug-like allosteric activators of GDE2. Downstream effectors of the GDE2-GPC6 cleavage pathway might be more amenable to therapeutic targeting, such as the RhoA-ROCK signaling cascade whose inhibition is known to mimic GDE2 activation in promoting neurite outgrowth.

In conclusion, the new findings establish GDE2 as a cell-autonomous inducer of neuroblastoma differentiation with prognostic value. Future studies should address how glypican release from the cell surface triggers transmembrane signaling events and, together with GDE structure-function studies, they may open new therapeutic possibilities for overcoming the differentiation-inhibited state of neuroblastoma cells.
